# Effect of Defects on the Mechanical and Thermal Properties of Graphene

**DOI:** 10.3390/nano9030347

**Published:** 2019-03-03

**Authors:** Maoyuan Li, Tianzhengxiong Deng, Bing Zheng, Yun Zhang, Yonggui Liao, Huamin Zhou

**Affiliations:** 1 State Key Laboratory of Material Processing and Die & Mold Technology, Huazhong University of Science and Technology, Wuhan 430074, China; limaoyuan@hust.edu.cn (M.L.); uezuuezu@hust.edu.cn (T.D.); zhengbing@hust.edu.cn (B.Z.); hmzhou@hust.edu.cn (H.Z.); 2 Key Laboratory of Material Chemistry for Energy Conversion and Storage, School of Chemistry and Chemical Engineering, Huazhong University of Science and Technology, Ministry of Education, Wuhan 430074, China; ygliao@mail.hust.edu.cn

**Keywords:** mechanical properties, thermal properties, graphene, defect, molecular dynamic

## Abstract

In this study, the mechanical and thermal properties of graphene were systematically investigated using molecular dynamic simulations. The effects of temperature, strain rate and defect on the mechanical properties, including Young’s modulus, fracture strength and fracture strain, were studied. The results indicate that the Young’s modulus, fracture strength and fracture strain of graphene decreased with the increase of temperature, while the fracture strength of graphene along the zigzag direction was more sensitive to the strain rate than that along armchair direction by calculating the strain rate sensitive index. The mechanical properties were significantly reduced with the existence of defect, which was due to more cracks and local stress concentration points. Besides, the thermal conductivity of graphene followed a power law of λ~*L*^0.28^, and decreased monotonously with the increase of defect concentration. Compared with the pristine graphene, the thermal conductivity of defective graphene showed a low temperature-dependent behavior since the phonon scattering caused by defect dominated the thermal properties. In addition, the corresponding underlying mechanisms were analyzed by the stress distribution, fracture structure during the deformation and phonon vibration power spectrum.

## 1. Introduction

Due to its excellent mechanical, thermal and other physical properties, Graphene (Gr) has attracted great attention from researchers since it was first prepared by Geim and Novoselov [1] in 2004. Experimental studies have proven that Gr presents superior thermal conductivity (TC) of ∼4840–5300 W/mK [2], which is far more than other common thermal management materials, such as copper (~400 W/mK), silver (~429 W/mK), etc. [3]. By nanoindentation in an atomic force microscopy, the Young’s modulus and fracture strength of Gr are reported as 1.0 ± 0.1 TPa and 123.5 GPa [4], respectively. The outstanding physics properties are mainly due to the two-dimensional structure consisting of *sp*^2^ bonded carbon atoms.

However, Gr fabricated by various experimental methods is not perfect and different types of defects are unavoidable. The common types of defects include single vacancy (SV) [5,6], double vacancy (DV) [5,6], Stone–Wales (SW) [7], grain boundaries [8], carbon adatoms [9], etc. The pristine structure would be destroyed by the existence of defects, which have a significant impact on the mechanical and thermal properties of Gr. For instance, by using Raman spectral, Zandiatashbar et al. [10] found that the Young’s modulus of Gr was maintained and fracture strength decreased by only ~14% even at a high concentration of sp^3^-type defects; however, it decreased significantly with the existence of vacancy defect. Using the molecular dynamic (MD) simulations, Mortazavi et al. [11] indicated that the TC of Gr decreased ~50% at a defect concentration of ~0.25%, and the Young’s modulus, fracture strength and fracture strain decreased with the increase of defect concentration. Zhao et al. [12] found that the oxygen plasma treatment could reduce the TC of Gr significantly at a low defect concentration (~83% reduction for ~0.1% defect concentration) through MD simulations and non-contact optothermal Raman measurement. Jing et al. [13] indicated that the vacancy defect could decrease the Young’s modulus while the reconstruction of vacancy could stabilize the modulus. Although there are some pioneering reports on the mechanical and thermal properties of Gr, as described above, there is still a lack of comprehensive study about some important factors, e.g., how the type and concentration of defects are related to the mechanical and thermal properties, especially at different temperatures. The reduction or enhancement mechanisms for the TC of defective Gr at different temperature were also not well understood. Meanwhile, the Gr is commonly used as nanofiller to enhance the mechanical and thermal properties of polymer materials, e.g., Gr/epoxy nanocomposites [14,15], etc., thus it is of great significance to fully understand the mechanical and thermal properties of Gr and effects of various defects.

In this study, we conducted a series of MD simulations to investigate the mechanical and thermal properties of Gr. The effects of temperature and strain rate on the Young’s modulus, fracture strength and fracture strain were first studied. Three typical defects, SV, DV and SW, were considered in detail. Besides, the influences of temperature, system size and defects on the TC of Gr were investigated by non-equilibrium molecular dynamic (NEMD) simulations. By calculating the phonon vibration power spectrum and phonon scattering, the related mechanisms of TC of Gr with/without defect at different temperature were clarified.

## 2. Computational Methods

### 2.1. Molecular Model of Gr

**Figure 1:**
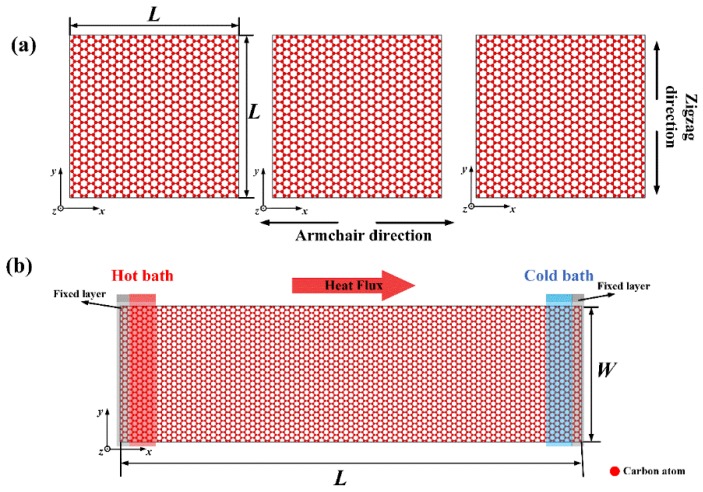
Molecular model of: (**a**) the armchair and zigzag Gr for uniaxial tensile test; and (**b**) Gr for the calculating TC.

MD simulations were conducted to evaluate the mechanical and thermal properties of Gr, and the molecular models were first constructed. The molecular model of Gr for the uniaxial tensile test consisted of 960 carbon atoms with the dimension around 50 Å × 50 Å, which has been proven to successfully calculate the mechanical properties of Gr [16], as shown in Figure 1a. Figure 1b shows the molecular model for calculating the TC of Gr with a size of 59 Å × 200 Å containing 4512 carbon atoms.

**Figure 2:**
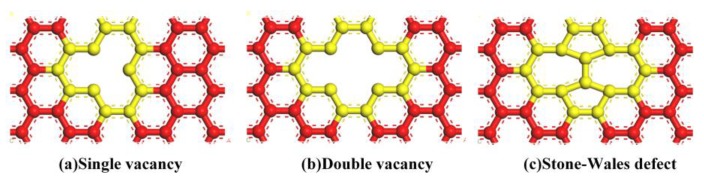
Types of defect studied in this work: (**a**) Single vacancy (SV); (**b**) Double vacancy (DV); and (**c**) Stones–Wales (SW).

To investigate the effect of different defects, Gr with three common types of defects, namely SV, DV and SW, was constructed (as shown in Figure 2). The SV and DV were created by removing one carbon atom or two adjacent carbon atoms from the pristine Gr, respectively. The SW was created by rotating one of the C–C bonds by 90°. Due to the different sizes of the models, the defect concentration varied from 0% to 3.125% for calculating mechanical properties, and from 0% to 0.24% in the study of TC. The defect concentrations of SV and DV were defined as the number density of atoms removed from the pristine Gr. The concentration of SW was defined by considering two defective atoms for each defect. The three kinds of defects were randomly distributed on the Gr sheets, respectively.

All MD simulations were conducted by The Large-scale Atomic/Molecular Massively Parallel Simulator (LAMMPS) [17], and the velocity-Verlet method was used to integrate the equations of motion. The adaptive intermolecular reactive bond order (AIREBO) [18] potential was used to simulate the C–C interaction, as it has successfully investigated the thermal and mechanical properties of carbon-based system, e.g., Gr [16], graphene [15], etc. A time step of 1 fs was used in the whole stages.

### 2.2. Calculation of Mechanical and Thermal Properties

After the initial models were established, the mechanical properties were calculated by uniaxial tensile test. As shown in Figure 1a, the chirality of Gr was considered, i.e., the armchair (*x*) and zigzag (*y*) directions. To eliminate the boundary effect, the periodic boundary conditions were applied along the x and y directions. The system was first relaxed to reach an equilibrium state; the relaxation involved two steps. At the beginning, an energy minimization was performed using the conjugate gradient algorithm. The system was then relaxed in a canonical NVT ensemble (i.e., constant number of atoms, volume and temperature) at temperature T = 300 K for 10^6^ timesteps followed by a microcanonical NPT ensemble along x/y directions (i.e., constant number of atoms, pressure and energy) for another 10^6^ timesteps. Followed by the equilibration, a constant uniaxial strain was applied along the x- or y-direction with a strain rate of 5 × 10^−3^ ps^−1^. The atomic stress of the Gr during the uniaxial tension were calculated by the viral theorem using Equation (1) [19]:

(1) $$\sigma_{i}^{\alpha \beta }=\frac{1}{\Omega_{i}}\left\{-m_{i}v_{i}^{\alpha }v_{i}^{\beta }+\frac{1}{2}\sum_{j\neq i}F_{ij}^{\alpha }r_{ij}^{\beta }\right\}$$

where Ω*_i_*, *m_i_* and *v_i_* represent the volume, mass and velocity of atom *i*, respectively; *F_ij_* and *r_ij_* are the force and distance between atom *i* and *j*, respectively; and indices α and β denote the Cartesian coordinate components. The thickness of Gr was determined by van der Waals interaction between the single layers, i.e., 3.35 Å. Then, the mechanical properties including Young’s modulus, fracture strength and fracture strain could be obtained from the stress–strain curves.

In this study, the direct non-equilibrium molecular dynamic (NEMD) [20] was applied to calculate the TC. In the NEMD method, the atoms near the left/right end were treated as the heat/cold baths, the temperature of which was set to T_H_ = T_0_ (1 + Δ) and T_C_ = T_0_ (1 − Δ) by Langevin thermostat, respectively (as shown in Figure 1b). T_0_ is the average temperature and Δ is the normalized temperature difference. To evaluate the effect of temperature, T_0_ varied from 300 K to 900 K, while Δ was fixed at 0.03. During the NEMD simulations, the energies removed from the cold bath and added to the hot bath as a function of time were calculated. The sum of added/removed energy is equal to zero, thus the total energy is conserved. The heat flux along the x-direction *J_x_* can be expressed by:

(2) $$J_{x}=\frac{dE/dt}{A}$$

where *E* is the accumulated energy, *t* is the simulation time in NVE ensemble and *A* is the cross-section area obtained by the width multiplied by thickness. Once the steady-state temperature profile along the heat flux was reached, the TC could be calculated by Fourier law:

(3) λ=JxdTdx

where *λ* and *dT/dx* are the TC and temperature gradient along the *x* direction, respectively. After the temperature profile was stable, another 10 ns of NEMD simulations were applied. The final TC was the average value of the last ten different time blocks (every 10^6^ timesteps), the error bars were determined by the standard deviation.

## 3. Results and Discussion

### 3.1. Validation of Models

**Figure 3:**
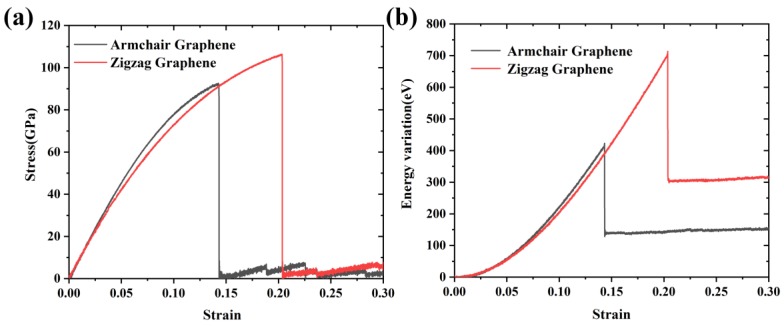
(**a**) Stress–strain curves for Gr along the armchair and zigzag directions; and (**b**) the total energy variations during the loading process.

To evaluate the molecular model and the AIREBO potential, the mechanical and thermal properties of pristine Gr were calculated. Figure 3 shows the stress–strain curves and the total energy variations along the armchair and zigzag directions. According to the stress–strain curves, the Young’s modulus could be calculated by linear fitting the curves when the strain <2%; the value of fracture strength was defined as the maximum stress while the corresponding strain was the fracture strain. The calculated results and the relevant values obtained by previous simulations or experiments are displayed in Table 1. It can be seen in Table 1 that the calculated results, including Young’s modulus (i.e., 961 GPa and 911 GPa for Gr along the armchair and zigzag direction, respectively), fracture strength (i.e., 93 GPa and 106 GPa for Gr along the armchair and zigzag directions, respectively) and fracture strain (0.14 and 0.20 for Gr along the armchair and zigzag directions, respectively) were in agreement with the experimental values [4] (i.e., ~1000 GPa, 130 ± 10 GPa, and 0.25). The simulation results were also consistent with previous studies [16,21,22], specific differences being determined by the selection of simulation conditions such as potentials, system size, etc.

**Figure 4:**
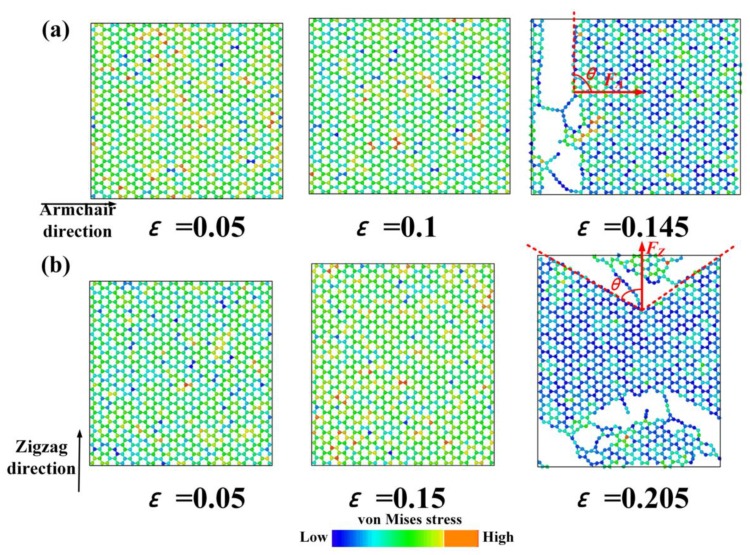
The fracture process and distributions of von Mises along: (**a**) the armchair direction and; (**b**) the zigzag direction.

The Young’s modulus along the armchair direction was less than that along the zigzag direction, while the fracture strength and strain were greater than those along the zigzag direction, which indicated a typical anisotropic behavior. The fracture process and the distribution of von Mises along the armchair and zigzag directions are shown in Figure 4. The von Mises was calculated as:

(4) $$\sigma_{m}=\sqrt{\frac{1}{2}\left[{\left(\sigma_{11}-\sigma_{22}\right)}^{2}+{\left(\sigma_{11}-\sigma_{33}\right)}^{2}+{\left(\sigma_{22}-\sigma_{33}\right)}^{2}+6(\sigma^{2}{}_{11}+\sigma^{2}{}_{22}+\sigma^{2}{}_{33})\right]}$$

where σ represents stress, and subscripts 1–3 represent the coordinate directions (i.e., x, y and z directions). The Gr was subjected to simple elongation deformation in the elastic deformation stage, and the carbon rings remained hexagon. As the load increased, the carbon ring at the boundary began to be irregular. When the load exceeded a certain value, Gr began to crack. For the armchair direction, the break of the C–C bonding occurred at the both sides of boundary and propagated inward rapidly. For the zigzag direction, the crack propagated rapidly and showed a remarkable zigzag shape. Comparing the fracture process, it was found that the fracture along the armchair direction belonged to Mode I (i.e., the tensile stress was perpendicular to the crack), while the fracture along the zigzag direction belonged to a mixture of Modes I and II (i.e., the angle between tensile stress and crack was ~60°). Based on the force analysis, the force parallel to the loading direction *F*_A_ was greater than that with an angle of 60° to the loading direction *F*_Z_, thus Mode I ruptured first, leading a greater fracture strength (106 GPa) and strain (0.20) along the zigzag direction than those (93 GPa and 0.14, respectively) along the armchair direction.

**Figure 5:**
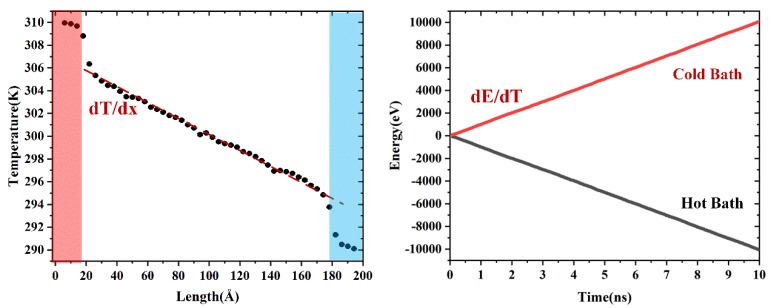
(**a**) Steady-state temperature profile of Gr without defect obtained using the NEMD simulations at 300 K. The color bars highlight the hot/cold bath in simulation. (**b**) Energies added to the hot bath and removed from the cold bath with respect to the time.

The TC of pristine Gr was also calculated. Figure 5 shows the steady-state temperature profile along the heat flux direction and the calculated energies added to the hot bath and removed from the cold bath according to the time. Based on Equations (2) and (3), the TC value of pristine Gr was 181.97 ± 0.007 Wm^−1^ K^−1^ at the temperature of 300 K. The measured value of TC with respect to time during the steady-state simulations is provided in the Supplementary Materials. The TC and relevant values obtained by simulations or experiments are listed in Table 2. The results show that the TC of Gr was related with the calculation method, system size, potentials, etc. The present results were consistent with the previous simulation results [24,25] (with same potential and size). Therefore, the above results confirmed the validity of the AIREBO potential, molecular models and calculation method.

### 3.2. Effects of Temperature and Strain Rate on the Mechanical Properties

**Figure 6:**
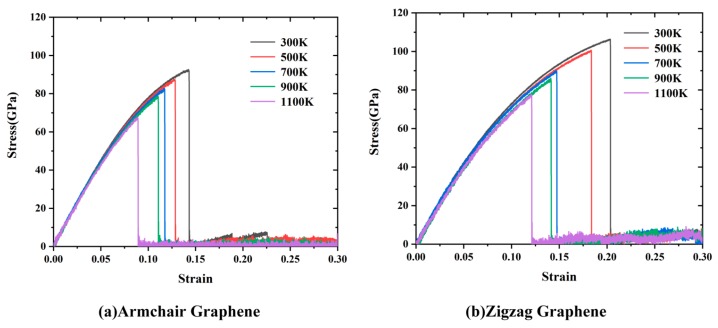
The stress–strain curves of Gr at different temperature along: (**a**) armchair; and (**b**) zigzag.

The temperature/strain rate-dependent effects are of great importance for mechanical properties of low-dimension materials, thus the effects of temperature and strain rate were investigated. The temperature varied from 300 K to 1000 K, and the stress–strain curves were along different directions, as shown in Figure 6. The curves indicated that the Gr showed a similar deformation behavior, i.e., a brittle fracture behavior, indicating the stable molecular structure of Gr.

**Figure 7:**
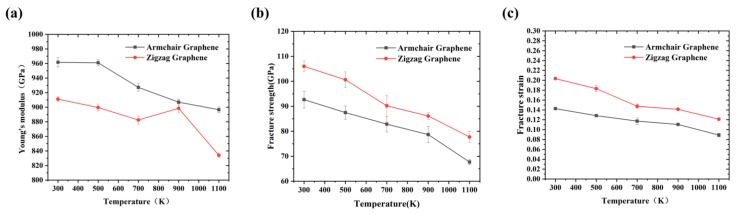
(**a**) The Young’s modulus; (**b**) the fracture strength; and (**c**) the fracture strain of Gr along the armchair and zigzag directions at different temperatures.

As shown in Figure 7a, the Young’s modulus, fracture strength and strain of Gr along different directions decreased with the increase of temperature. When the temperature increased from 300 K to 1100 K, the Young’s modulus along the armchair direction decreased ~6.8% (from 961.61 GPa to 896.56 GPa), fracture strength decreased ~27% (from 92.67 GPa to 67.68 GPa) and fracture strain decreased ~37.6% (from 0.14 to 0.089), while it decreased ~8.5% (from 911.08 GPa to 834 GPa), ~26.7% (from 106 GPa to 77.73 GPa) and ~40.49% (from 0.204 to 0.121) along the zigzag direction, respectively. The temperature-dependent mechanical properties along the different directions was similar. Compared with the fracture strength and strain, the Young’s modulus was less sensitive to temperature. The total kinetic energy increased with the increase of temperature; the thermal vibration of carbon atoms was more vigorous, leading a larger vibration amplitude around the equilibrium position. Under the action of external forces, the atoms were more likely to be away from their original equilibrium position, resulting in a softer and less rigid structure. Meanwhile, at the high temperature, in addition to the formation of cracks at the boundary, the cracks also occurred from inside, resulting in more defect cracks. Therefore, the fracture strength and strain were significantly reduced. Tang et al. [28] explained the mechanism from the perspective of energy: the deformation process of Gr was determined by the strain energy and thermal energy; the thermal energy increased with the temperature, thus the strain energy required for the fracture reduced.

**Figure 8:**
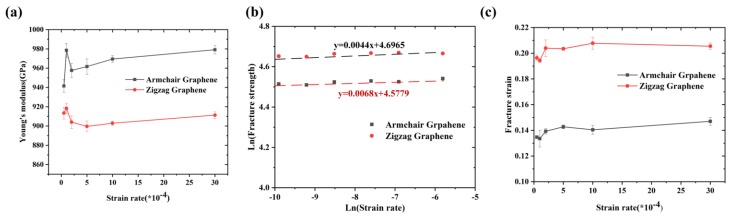
(**a**) The Young’s modulus; (**b**) the fracture strength; and (**c**) the fracture strain of Gr along the armchair and zigzag directions at different strain rates.

As shown in Figure 8, at different strain rate (0.5 × 10^−5^−3 × 10^−3^ ps^−1^), the Young’s modulus was insensitive to the strain rate, while the fracture strength and strain slightly increased at higher strain rate. The lower strain rate means a longer response time for Gr, which would increase the number of atoms that could overcome the energy barrier required for fracture, leading a lower fracture strength and strain. However, the effect of strain rate was less significant than that of temperature. The relation between fracture strength and strain rate can be described by Arrhenius equation [29]:

(5) $$\overset{•}{\varepsilon }=A\sigma^{\frac{1}{m}}\exp(-\frac{Q}{RT})$$

where $\overset{•}{\varepsilon }$, *σ, Q, R, T* and *m* represent the strain rate, fracture strength, the activation energy, universal gas consent, temperature and the strain-rate sensitivity, respectively, and *A* is a constant. By taking the natural logarithm of both sides of Equation (6):

(6) $$\ln(\overset{•}{\varepsilon })=\ln(A)+\frac{1}{m}\ln(\sigma )-\frac{Q}{RT}$$

At the constant temperature, the partial differentiation of Equation (7):

(7) $$m=\frac{\ln(\sigma )}{\ln(\overset{•}{\varepsilon })}$$

The strain-rate sensitivity *m* can be obtained from the slope of the ln (*σ*) versus ln ($\overset{•}{\varepsilon }$). The results show that the *m* along the armchair and zigzag directions were 0.0044 and 0.0068, respectively. Therefore, the fracture strength of Gr along the zigzag direction was more sensitive than to strain rate that along the armchair direction.

### 3.3. Effect of Defects on the Mechanical Properties

**Figure 9:**
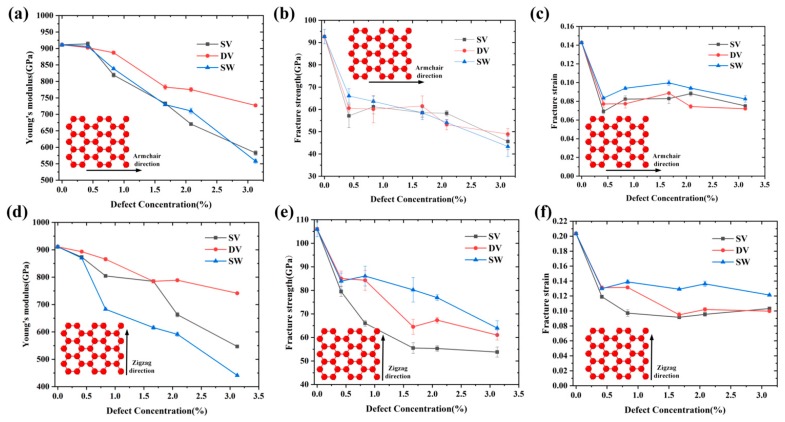
Under different defect types and concentrations, the variations of: (**a**) Young’ modulus; (**b**) fracture strength; and (**c**) fracture strain of Gr along the armchair direction. The variations of: (**d**) Young’ modulus; (**e**) fracture strength; and (**f**) fracture strain of Gr along the zigzag direction with respect to defect concentration at different defect types.

In this section, the effect of defects was investigated. As described above, three types of defects, i.e., SV, DV and SW, were considered. As shown in Figure 9, the mechanical properties of Gr decreased remarkably with the increase of defect concentrations, and it showed a similar behavior along different direction. For simplicity, only the mechanical properties of Gr along the zigzag are discussed. At the same defect concentration, Gr was more sensitive to the SV than DV. For example, when the concentration was 1.67%, the Young’s modulus of Gr containing SV decreased ~47.6% (from 106 GPa to 55.50 GPa), while it decreased ~39.1% (from 106 GPa to 64.53 GPa) for the Gr containing DV. The main reason was the fact that the SV produced more dangling bonding than DV, resulting in more cracks. The literature [13] confirms that, at the same missing carbon atoms, the mechanical properties of Gr are mainly determined by the number of dangling bonding. Zandiatashbar et al. [10] also quantitatively investigated the effect of defects on the mechanical properties using the Raman spectroscopy; their results show that the Young’s modulus and strength of Gr is insensitive to *sp*^3^-type defect, while decreases significantly with the increase of vacancy defect. Meanwhile, further increase of defect concentration has little effect on the fracture strength and strain.

**Figure 10:**
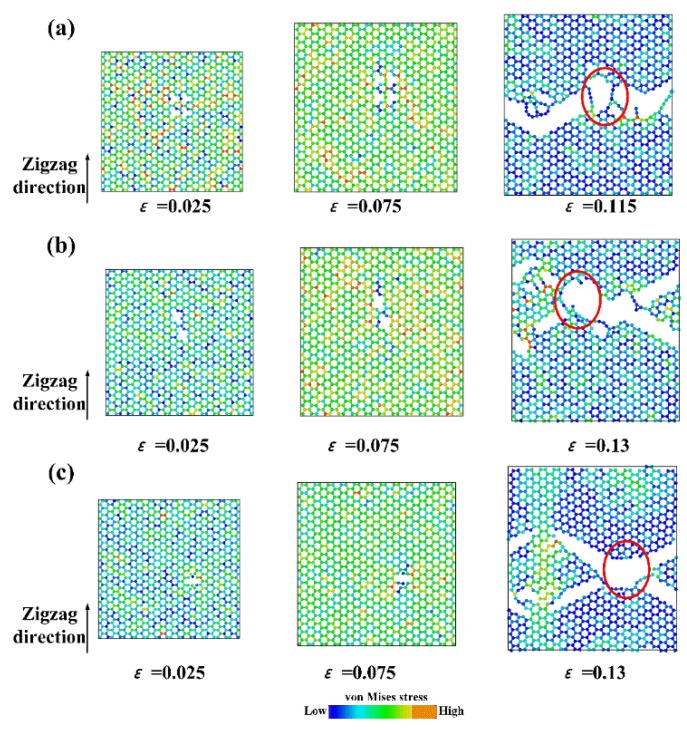
The fracture process of Gr containing: (**a**) SV; (**b**) DV; and (**c**) SW. The red ring indicates the location of defect.

Figure 10 shows the fracture process of Gr containing different defects. Compared with the fracture process of pristine Gr, the overall trend was similar for the defective Gr. However, the initial crack did not occur near the boundary, but rather formed inside and gradually spread until the fracture. Before a crack occurs, the existence of defects will cause the local stress concentration point, leading to a lower fracture strength. Due to the limited size (50 Å × 50 Å) and high temperature (>300 K) in present study, the defect concentration was limited since the structure with high defect concentration was unstable at high temperature. When the defect concentration was larger, the interaction between defects, including the merging of defect and crack blocking, could result in more complicated fracture behavior. For example, Xu et al. found that when the concentration exceeded ~7%, *sp-sp*^2^ and *sp*^2^*-sp*^3^ network structures were formed, and fracture behavior for Gr changed from brittle to ductile.

### 3.4. Effects of Temperature and System Size on the Thermal Properties

**Figure 11:**
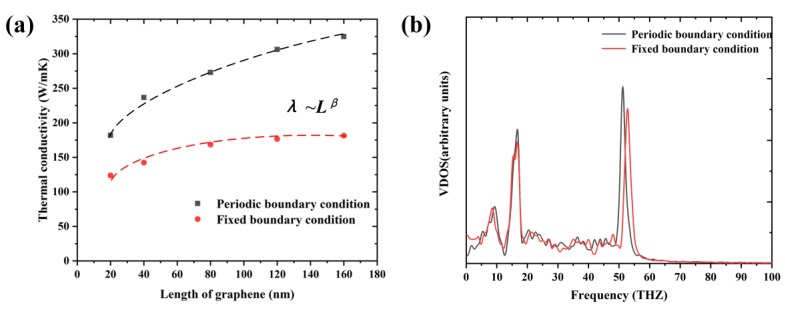
(**a**) The variation of TCs of pristine Gr with respect to length; and (**b**) the VDOS of Gr under periodic/fixed boundary conditions.

The thermal properties of Gr and other low-dimension materials showed significant relationship with temperature and system size. As shown in Figure 11, the TCs of Gr with lengths of 20, 40, 80, 120 and 160 nm were calculated. The results indicate that the TC of Gr increased monotonically with increase of length. By fitting the data, the TC was found to follow the power law of λ~*L*^0.28^, which was similar to the previous results of λ~*L*^0.35^ obtained by Guo et al. [30]. Such phenomenon was also consistent with the previous studies about the carbon nanotube [31], λ~*L^β^* and *β*~0.3–0.4. The size-dependent behavior was due to the long mean free path of phonons (MFP) of Gr, ~775 nm [32], which was much longer than that used in MD. Therefore, apart from the phonon–phonon scattering, the phonon scattering existed at the boundary of Gr. With the increase of length, more phonons will be excited and contribute to the increase of TC. We also compared the TCs calculated at different boundary conditions along the width direction (y), as shown in Figure 11a. The fixed boundary condition means that the particle could not interact across the boundary and move from one side of the box to the other, i.e., the length of Gr was finite in the width direction. This clearly indicated that the TCs obtained at the fixed boundary condition were smaller than those obtained at the periodic boundary condition. For instance, when the length was 80 nm, the TCs at the periodic and fixed boundary condition were 273.1 and 168.6 W/mK, respectively. At the fixed boundary condition, the power law between the TCs and length, λ~*L*^0.18^, the index of power law was smaller than that at the periodic boundary condition. This was because, at the periodic boundary condition, the phonons can across the regions perpendicular to the direction of the heat flow without boundary scattering, thus the width of Gr had negligible effect on the calculation results. In addition, the vibration density of states (VDOS) of Gr at the two different boundary conditions were calculated. The VDOS can be obtained by calculating the Fourier transformation of atomic velocities autocorrelation function at the equilibrium state:

(8) $$D\left(\omega \right)=\int_{0}^{\tau }\Gamma \left(t\right)\cos\left(\omega t\right)dt$$

where *ω* is frequency, *D*(*ω*) is vibration density of state at frequency *ω*, *τ* is the total time, and Γ(t) is the velocity autocorrelation function of atoms, which is given by:

(9) Γ(t)=〈v(t)⋅v(0)〉

where *v(t)* is the atom velocity at time *t*, and <···> denotes time and atom number-averaged velocity autocorrelation function. In this study, the velocity was correlated every 5 fs with a total integration time *τ* = 25 ps. Compared with the periodic boundary condition, the VDOS of Gr was suppressed at the fixed boundary condition, especially at the high frequency (~50 THZ), leading to a lower TC.

**Figure 12:**
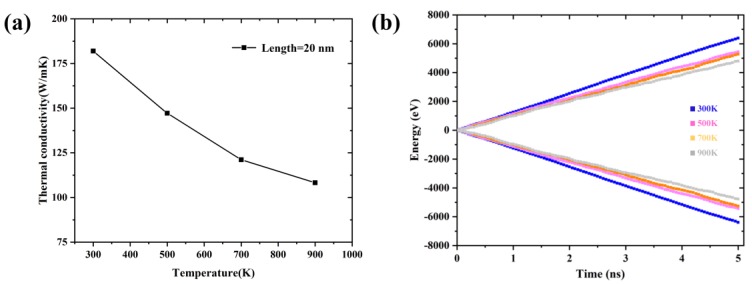
(**a**) The TCs of Gr; and (**b**) the energies added to the hot bath and removed from the cold bath during the NEMD at different temperature.

The TCs of Gr at different temperature are shown in Figure 12a. The TCs of Gr decreased with increase of temperature, consistent with previous results obtained by Hu et al. [33]. Notably, Seol et al. [34] found that, at the low temperature (<300 K), the lattice vibration increased with the increase of temperature, leading to a longer MFP and higher TC. As the temperature continued to rise, the TC decreased. We also calculated the energies added to the hot bath and removed from the cold bath at different temperatures and the accumulated energy with respect to simulation times, i.e., the corresponding heat flux decreased with the increase of temperature. At high temperature, the phonon motion became more vigorous and the interaction and collision between phonons increased, resulting in stronger inelastic phonon scattering and lower TC.

### 3.5. Effect of Defects on the Thermal Properties

**Figure 13:**
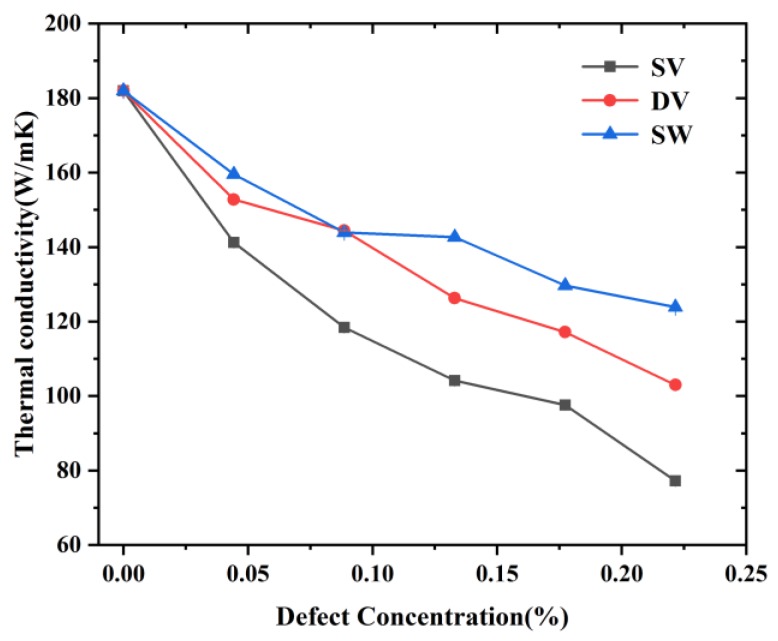
The TCs of Gr with different concentration at different types of defects.

The effect of defects, including SV, DV and SW, on the TC of Gr were investigated. As shown in Figure 13, the TCs of Gr decreased with the increase of defect concentration. When the defect concentration varied from 0% to 0.24%, the TCs of Gr containing SV, DV and SW varied from 181.97 to 77.21 W/mK (~57.6%), 102.97 W/mK (~43.4%) and 123.86 W/mK (~31.9%), respectively. This agreed with the previous simulation results of TCs of Gr obtained by Mortazavi et al. [11] and that of carbon nanotube obtained by Sevik [35]. Zhao et al. [12] also found that the defect introduced by oxygen-plasma treatment could decrease significantly the TC of Gr (~83% reduction at a defect concentration of ~0.1%) using the non-contact optothermal Raman technique. The results indicate that the TC of Gr decreased even at a low defect concentration (~0.24%), which was mainly due to the scattering caused by defect. Based on the theory of classical TC, the TC can be obtained by:

(10) $$\lambda =\frac{1}{3}CVl$$

where *C*, *V* and *l* denoted the specific heat capacity, group velocity of sound wave in solid and the MFP, respectively. The MFP of pristine Gr was determined by the phonon–phonon scattering. While the defects were introduced, the effective MFP was:

(11) $$\frac{1}{l}=\frac{1}{l_{phonon-phonon}}+\frac{1}{l_{defect-phonon}}$$

where *l_phonon-phonon_* and *l_defect-phonon_* are the length of phonon–phonon scattering and scattering caused by defects, respectively. According to Equations (10) and (11), the TC of defective Gr can be obtained:

(12) $$\frac{1}{\lambda }\propto \frac{1}{l}\propto \frac{1}{l_{phonon-phonon}}+\frac{1}{l_{defect-phonon}}$$

According to Equation (12), the existence of defects could decrease the MFP of pristine Gr, resulting in a lower TC. Comparing different defect types, the TC of Gr containing SV was the smallest (a reduction of ~57.6%) at the same defect concentration. Such phenomenon can be explained as follows. At the same concentration, the Gr containing SV had more defect scatters than the Gr containing DV, resulting in more phonon defect scattering [24]. Meanwhile, a SV was formed by removing one atom leaving three carbon atoms two-coordinated, effectively breaking the *sp*^2^ structure of the local lattice, while a DV was formed by removing two adjusted atoms as the local structure can rearrange to restore the three-coordinated *sp*^2^ bonding by creating an octagon and two pentagon structures [35]. The two-coordinated atoms were less stable, leading to higher level of defect scattering. Previously, Haskin et al. [35] found that the Gr containing SV decreased ~80% at a concentration of 0.1%, while the Gr containing DV and SW decreased ~70%. Zhang et al. [24] also found that different types of defects had different effects on TC at a low concentration (≤0.2%). When the concentration increased (>0.2%), they had similar effect on TC, which was mainly because the heat transport mechanism changed from propagating to diffusive, and the TC was insensitive to the defect type in diffusive form.

**Figure 14:**
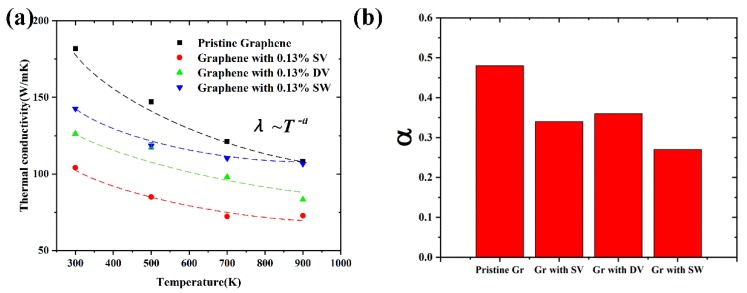
(**a**) The TCs of Gr with/without defect at different temperature; and (**b**) the corresponding power law index *α*.

Next, we investigated the TCs of Gr with/without defect at different temperatures. The TCs of Gr containing different defects at different temperatures are shown in Figure 14a, at the concentration of 0.13%. It indicated that the TCs of Gr with/without decreased with the increase of temperature. For instance, when the temperature increased from 300 K to 900 K, the TCs of Gr containing SV, DV and SW varied from 104.12 to 72.82 W/mK (~30%), 126.28 W/mK to 83.26 W/mK (~34%) and 142.65 W/mK to 106.53 W/mK (~25%), respectively. The TCs were fitted by power law, *λ*~*T^−α^*; the corresponding index is shown in Figure 14b. Compared with the pristine Gr, the power law index of defective Gr decreased significantly, i.e., showing a weak temperature-dependent behavior. The main reason was that, compared with the phonon scattering caused by temperature, the scattering caused by defects was the dominant factor for the TC of defective Gr, which was also consistent with previous results obtained by Zhang [24] and Hu et al. [33].

## 4. Conclusions

In summary, we investigated the mechanical and thermal properties of Gr by conducting MD simulations. For the mechanical properties, the effects of temperature, strain rate and defects were studied. The results show that the mechanical properties, including Young’s modulus, fracture strength and fracture strain, decreased with the increase of temperature. By calculating the strain rate sensitivity index, it was found that the mechanical properties of Gr along the zigzag direction were more sensitive to strain rate than those along the armchair direction. Then, the existence of defects, including SV, DV and SW, was found to significantly reduce the mechanical properties, which were more sensitive to SV than DV at the same concentration. Meanwhile, the local stress point caused by defects reduced the fracture strength.

For the thermal properties, the effects of temperature, system and defect were investigated. It was found that the TC followed a power law of λ~*L*^0.28^: more phonons were excited with the increase of length, contributing to the TC. The results also indicate that the TC of Gr containing SV, DV and SW decreased ~57.6%, ~43.4% and ~31.9% even at the low concentration of 0.23%, respectively, which was mainly due to the reduced MFP caused by defect scattering. Besides, the TCs of Gr with/without defect at different temperature were calculated, showing that, compared with the phonon scattering caused by temperature, the phonon scattering caused by defect dominated the thermal properties. The TCs of defective Gr showed a low temperature-dependent behavior. The above findings can provide a comprehensive understanding in the mechanical and thermal properties of Gr.

## Figures and Tables

**Table 1:** The calculated results and relevant values obtained by previous simulations or experiment.

References	Method	Direction	Young’s Modulus (GPa)	Fracture Strength (GPa)	Fracture Strain
Lee (2008) [4]	Experiment	/	1000	130 ± 10	0.25
Liu (2007) [23]	DFT	Armchair	1050	110	0.19
		Zigzag	1050	121	0.26
Q.X. Pei (2010) [16]	MD (AIREBO)	Armchair	890	105	0.17
		Zigzag	830	137	0.27
Ansari (2012) [21]	MD (Tersoff)	Armchair	790	123	0.23
		Zigzag	807	127	0.22
This paper	MD (AIREBO)	Armchair	961	93	0.14
		Zigzag	911	106	0.20

**Table 2:** The TC and relevant values obtained by simulations or experiment.

References	Potentials	Method	Size	TC at 300 K (Wm^−1^ K^−1^)
Balandin (2008) [2]	/	Experiment	~0.5–1 *u*m	~4840–5300
Wei (2011) [25]	AIREBO	RNEMD	102 × 102Å^2^	77.3
Yang (2012) [26]	AIREBO	EMD	(90~270) × (40~180) Å^2^	~3200–5200
Xu (2014) [27]	Tersoff	NEMD	50 × (2 × 150) Å^2^	~400–1800
Zhang (2012) [24]	AIREBO	RNEMD	61 × 200 Å^2^	~170
This paper	AIREBO	NEMD	60 × 200 Å^2^	182

## Supplementary Materials

The following are available online at https://www.mdpi.com/2079-4991/9/3/347/s1.
