# Supplementary material for: Effect of Defects on the Mechanical and Thermal Properties of Graphene
Source: Nanomaterials (Basel). 2019 Mar 3;9(3):347. doi: 10.3390/nano9030347 (PMC6474032; doi:10.3390/nano9030347)
Supplement: Supplementary file 1 [file nanomaterials-09-00347-s001.pdf]

Supplementary Information

Effect of Defects on the Mechanical and Thermal Properties of Graphene

Maoyuan Li [1], Tianzhengxiong Deng [1], Bing Zheng [1], Yun Zhang [1,*], Yonggui Liao [2] and Huamin Zhou [1]

[1] State Key Laboratory of Material Processing and Die & Mold Technology, Huazhong University of Science and Technology, Wuhan 430074, Hubei, China; limaoyuan@hust.edu.cn (M.L.); uezuuezu@hust.edu.cn (T.D.); zhengbing@hust.edu.cn (B.Z.); hmzhou@hust.edu.cn (H.Z.)

[2] Key Laboratory of Material Chemistry for Energy Conversion and Storage, School of Chemistry and Chemical Engineering, Huazhong University of Science and Technology, Ministry of Education, Wuhan 430074, Hubei, China; ygliao@mail.hust.edu.cn (Y.L)

* Correspondence: marblezy@hust.edu.cn; Tel.: +86-27-87543492

I. The LAMMPS script for calculating thermal and mechanical properties of Gr

a. Uniaxial tensile test

```
units       metal
dimension 3
boundary p p s
atom style full
read_data graphene. Data
pair_style        airebo   3.0   1 1
pair_coeff        * * CH.airebo   C
neighbor  3.0 nsq
neigh_modify     delay   0       every 1 check yes
variable temperature equal 300
variable tstep equal 0.001
variable pressure equal 0
variable thermalstep equal 100
variable dumpstep equal 50000
variable relaxtime equal 200000
variable totaltime equal 500000
variable deformrate equal 0.0005
variable strain_max equal 0.3
variable total_runs equal round(v_strain_max/(v_tstep*v_deformrate))
velocity all create ${temperature} 1000000 rot yes dist gaussian
min_style      cg
minimize       1.0e-14 1.0e-14 10000 10000
unfix 1
fix      relax all npt temp ${temperature} ${temperature} 0.05 x 0 0 5 y 0 0 5 drag 0.2
dump      config all custom 100000 relax.*.lammpstrj id type x y z
thermo 1000
thermo_style      custom step temp etotal pxx pyy pzz press
restart       100000 relax.*.restart
timestep ${tstep}
run     ${relaxtime}
```

```
unfix relax
undump config
reset_timestep 0
variable toval equal ly*lx*3.35
variable vol equal ${toval}
variable vatom equal v_vol/v_nums
compute 1 all stress/atom NULL
variable total equal etotal
variable pair equal epair
variable bond equal ebond
variable angle equal eangle
variable dihedral equal edihed
compute xalls all reduce sum c_1[1]
variable xstress equal (c_xalls)/(v_toval*10000)
compute yalls all reduce sum c_1[2]
variable ystress equal (c_yalls)/(v_toval*10000)
compute zalls all reduce sum c_1[3]
variable zstress equal (c_zalls)/(v_toval*10000)
timestep        ${tstep}
thermo          2000
fix avestress all ave/atom 1 ${dumpstep} ${dumpstep} c_1[1] c_1[2] c_1[3] c_1[4] c_1[5] c_1[6]
variable            tmp equal "lx"
variable            L0 equal ${tmp}
variable            strain equal "(lx - v_L0)/v_L0"
variable            Cumulativels equal "(lx - v_L0)/10"
fix Step all print 100 "${strain} ${Cumulativels} ${xstress} ${total} ${bond} ${pair} ${angle} ${dihedral} “file grapoten.txt screen no
dump 2 all custom ${dumpstep} relax.*.lammpstrj id type x y z f_avestress[1] f_avestress[2] f_avestress[3] f_avestress[4] f_avestress[5] f_avestress[6]
fix 1 all nvt temp ${temperature} ${temperature} 0.05
fix   3 all deform 1 x erate ${deformrate} units box remap x
fix   4 all ave/time 2 500 1000 v_xstress v_ystress v_zstress file pressure.out
thermo_style custom step pe ke etotal lx ly lz v_strain v_xstress
run   ${total_runs}
```

b. NEMD for calculating TC

```
log $nlammps.log
units       metal
variable    T equal 300
variable    dt equal 0.0005
variable    th equal 310
variable    tl equal 290
variable    kB equal 1.3806504e-23
variable    eV2J equal 1.602763e-19
variable    A2m equal 1.0e-10
variable    ps2s equal 1.0e-12
atom_style full
dimension    3
boundary     f p s
neighbor 0.3 nsq
read_data graphene.data
```

```
pair_style        airebo      3.0    1    1
pair_coeff        * *         CH.airebo         C
neighbor                3.0 nsq
neigh_modify            delay 0        every 1 check yes
variable xlength equal "lx"
variable L1 equal ${xlength}
variable dx equal ${L1}/50
variable xlo equal "xlo"
variable xh equal ${xlo}
variable xl equal ${xh}+${L1}
variable xf1 equal ${xh}+${dx}
variable xf2 equal ${xl}-${dx}
variable xh1 equal ${xh}+${dx}
variable xh2 equal ${xh}+${dx}*5
variable xc2 equal ${xl}-${dx}*5
region rgbhigh block INF ${xf1} INF INF INF INF units box
region rgblow block ${xf2} INF INF INF INF INF units box
group gbhigh region rgbhigh
group gblow region rgblow
fix 2 gbhigh setforce 0 0 0
fix 3 gblow   setforce 0 0 0
region      hot      block ${xh1} ${xh2} INF INF INF INF units box
compute Thot all temp/region hot
region cold      block     ${xc2} ${xf2} INF INF INF INF units box
compute Tcold all temp/region cold
region          1      block ${xf1} ${xf2} INF INF INF INF units box
compute Tbetween all temp/region 1
group       between     region      1
timestep        ${dt}
thermo           100000
compute         ke all ke/atom
variable temp atom    c_ke*${eV2J}/(1.5*${kB})           ####    T
variable     tempatom atom c_ke*${eV2J}*2/3/${kB}
velocity between create ${T} 3$n16$n8 mom yes rot yes dist gaussian
min_style cg
minimize 1e-25 1e-25 5000 10000
fix     111     between nvt temp ${T} ${T} 0.05
timestep        ${dt}
thermo           10000
thermo_modify lost warn
run 1000000
write_restart $nrestart.1
unfix         111
fix nve between nve
run 1000000
write_restart $nrestart.2
unfix nve
fix           1 between nve
compute cc1 between chunk/atom bin/1d x lower 0.02 units reduced
fix cc1 between ave/chunk 10 100000 1000000 cc1 v_temp file $ntemp.profile1 ave running
fix      hot all langevin ${th} ${th} 0.025 59$n80$n4 tally yes
fix     cold all langevin ${tl} ${tl} 0.025 2$n859$n2 tally yes
```

```
fix_modify hot temp Thot
fix_modify cold temp Tcold
run 2000000
unfix cc1
unfix hot
unfix cold
unfix 1
write_restart $nrestart.data
reset_timestep 0
fix 1 between nve
fix hot all langevin ${th} ${th}    0.025 59$n80$n4 tally yes
fix cold all langevin ${tl} ${tl} 0.025 2$n859$n2 tally yes
fix_modify hot temp Thot
fix_modify cold temp Tcold
compute cc2 between chunk/atom bin/1d x lower 0.02 units reduced
fix cc2 between ave/chunk 10 100000 1000000 cc2 v_temp file $ntemp.profile2 ave running
fix e_exchange all ave/time 100000 1 100000 f_hot f_cold file $ne_exchange.dat
run 20000000
```

II. The measured value of TC during steady-state simulation

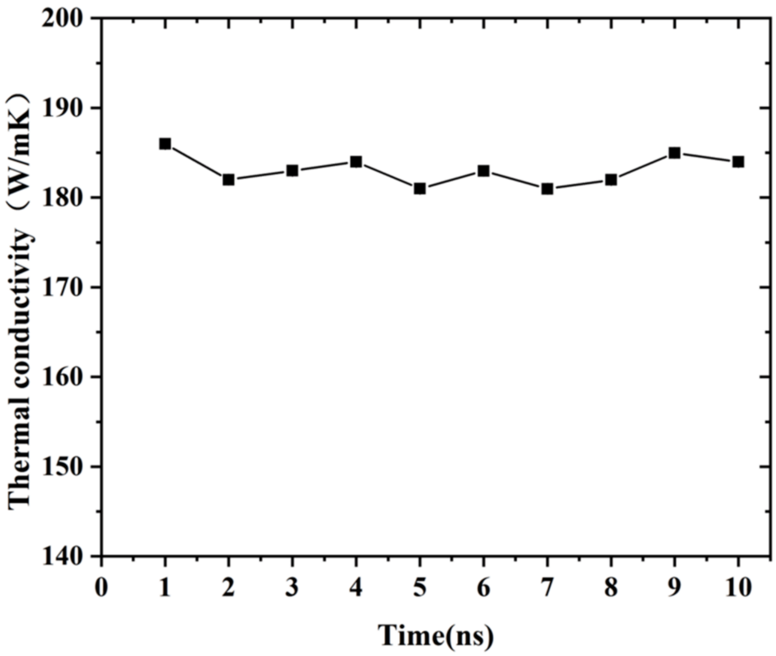

Figure S1. The measured value of TC with respect to simulation time during steady-state simulation.
